# Testicular membrane lipid damage by complex mixture of leachate from municipal battery recycling site as indication of idiopathic male infertility in rat

**DOI:** 10.2478/intox-2013-0028

**Published:** 2013-12

**Authors:** Jacob K. Akintunde, Ganiyu Oboh, Akintunde A. Akindahunsi

**Affiliations:** 1 Functional Foods, Nutraceuticals and Phytomedicine Research Laboratory, Department of Biochemistry, Federal University of Technology, Akure, Nigeria; 2 Department of Biosciences and Biotechnology, Biochemistry unit, College of Pure and Applied Sciences, Kwara State University, Malete, Nigeria

**Keywords:** EOMABRL, mixed-metal exposure, lipid membrane, infertility, rat

## Abstract

Leachate from a municipal battery recycling site is a potent source of mixed-metal released into the environment. The present study investigated the degree at which mixed-metal exposure to the municipal auto-battery leachate (MABL) and to the Elewi Odo municipal auto-battery recycling site leachate (EOMABRL) affected the lipid membrane of the testes in *in vitro* experiment. The results showed elevated level of mixed-metals over the permissible levels in drinking water, as recommended by regulatory authorities. In the leachate samples, the levels of malondialdehyde (MDA), a biomarker of lipid damage, was significantly (*p*<0.05) increased in rat testes in a dose-dependent manner. MDA induced by the municipal auto-battery leachate (MABL) was significantly (*p*<0.05) higher than the leachate from Elewi Odo municipal auto-battery recycling site (EOMABRL). The testicular lipid membrane capacity was compromised following treatment with leachate from the municipal battery recycling site, implicating mixed-metal exposure as the causative agent of testicular damage and male infertility.

## Introduction

The disorders of reproduction and hazards to reproductive health and associated functions have become prominent issues in recent decades after reports on adverse effects of certain chemicals. The male reproductive system is vulnerable to the effects of these chemicals, particularly because sensitive events take place during spermatogenesis. Industrialization and overgrowing urbanization are also suspected as causes of human exposure to different toxic chemicals. They may compromise the male reproductive system and produce cellular impairment both at structural and functional level (Beckman *et al.,* 1990; Godwin *et al.,* 2006). Xenobiotics alter the structure of the cell membrane by stimulating the lipid peroxidation process with ensuing complex sequences of biochemical reactions. Spermatozoa are rich in polyunsaturated fatty acids and are susceptible to membrane lipid peroxide ion (Muanya *et al.,* 2008; Lamirande *et al.,* 1992; Rosseli *et al.,* 1995; Sikka 1995, 1996).

The consumption of batteries has increased sharply in the last 30 years because of the versatility, low maintenance, reduced cost and the high requirements of the electronics industry (De Souza *et al.,* 2001). Disposal of spent batteries represents growing environmental challenges due to the metallic content, considered as hazardous waste (Sayilgan *et al.,* 2009). The batteries are used in radios, recorders, toys, remote controls, watches, calculators, cameras, laptop computers, camcorders and in many other objects where small quantities of power are required (Sayilgan *et al.,* 2009). It is estimated that the Zn-Mn batteries occupy over 90% of the total annual sales of portable batteries due to their low prices, especially in developing countries like Nigeria and China. They are usually rapidly run out and thrown away (Bartolozzi, 1990). As a special residue containing heavy metals, the waste batteries cause a serious concern due to their toxicity, abundance and permanence in the environment (Li & Xi, 2005). The recycling processes should be environmentally friendly and pose no adverse effects on biological organisms.

Leachate from a municipal battery recycling site (EOMABRL) may be implicated as a source of mixed-metals which, when indiscriminately dumped or improperly recycled, might have access to water bodies and food chains. They may consequently get into humans by drinking water or by ingestion and/or inhalation and thus induce toxic effects on the testes. To date, there are few or no reports on the effects of mixed-metal exposure on the testicular membrane of either humans or terrestrial animals. It is therefore imperative to determine the degree at which mixed-metal exposure to battery recycling site leachate (EOMABRL) affects the lipid membrane of the testes so as to induce necessary preventive and curative measures to prevent pathological conditions.

## Materials and methods

### Sampling site and leachate preparation

The sampling site, Elewi Odo municipal battery recycling site, is located on the Ibadan Northern part of Oyo State of Nigeria (latitude7°25.08'N and 7°25.11'N and longitudes 3°56.45'E and 3°56.42'E). The site is largely used for auto-battery waste recycling activities. It is at the back of a stream in the residential area. It covers about 2 acres of land. A randomized sampling technique (Houk, 1992; Siddique *et al.,* 2005) was employed to collect the first layer solid soils (0–15 cm deep) from five different points on the municipal auto-battery recycling site. At least five randomly collected samples from each site were pooled to make a single representative sample. The sample was air-dried, finely ground with a mortar and pestle, and sifted through a 63-µm (pore size) sieve to obtain a homogenous mixture.

Leachate (100%) was prepared from the homogenous mixture according to a standard procedure (ASTM, 1992; Ferrari *et al.,* 1999). Briefly, 100 g of the sample (homogenous mixture) was added to 100 ml of distilled water (w/v) and shaken for 48hr at (30±1) °C. After shaking, the sample was allowed to settle for 30 min to sediment visible particles, and then the supernatant was filtered with a 2.5 µm filter (Whatman No. 42) to remove the suspended particles. Finally, the sample was stored at 4 °C until use. It was designated as Elewi-Odo municipal auto-battery recycling site leachate (EOMABRL). Sample water was collected from the stream near the site (STREAM).

### Auto-battery leachate sampling and preparation

Eight different auto-batteries were randomly selected from different workshops of the town Ibadan in south-west of Nigeria. The liquids (*i.e.* the electrolyte in contact with the lead plates) and the lead plates of the battery were removed. The remaining wet dust of each battery was air-dried, finely ground with a mortar and pestle, and sifted through a 63-µm (pore size) sieve to obtain a homogenous mixture.

From each homogenous mixture, leachate (100%) was prepared according to a standard procedure (ASTM, 1992; Ferrari *et al.,* 1999). Briefly, 100 g of the sample (each homogenous mixture) was added to 100ml of distilled water (w/v) and shaken for 48hr at (30±1) °C. After shaking, the sample was allowed to settle for 30min to sediment visible particles and then the supernatant was filtered with a 2.5 µm filter (Whatman No. 42) to remove the suspended particles. Finally, the samples were stored at 4 °C until use. The samples were designated as BL1, BL2, BL3, BL4, BL5, BL6, BL7 and BL8. They are collectively regarded as municipal auto-battery leachate (MABL).

### Heavy metal analysis

The heavy metal levels of EOMABRL, MABL and STREAM were determined in accordance with standard methods (Federal Environmental Protection Agency; FEPA, 2001), (United States Environmental Agency; USEPA, 1996) and (World Health Organisation; WHO, 1992). Eleven metals, namely copper (Cu), lead (Pb), cadmium (Cd), cobalt (Co), chromium (Cr), molybdenum (Mo), selenium (Se), zinc (Zn), iron (Fe), nickel (Ni) and manganese (Mn) were analyzed in each leachate sample. Briefly, 100ml of each leachate was digested by heating with concentrated HNO_3_ and the volume was reduced to 2–3 ml. This volume was made up to 10 ml with 0.1 N HNO_3_ and the concentrations of the metals were determined using atomic absorption spectrophotometer (AAS) (AOAC, 1990). The levels of these metals were assessed because of their reported occurrence in both solid and liquid wastes in Nigeria (Longe & Balogun, 2010; Longe & Enekwechi, 2007; Farombi *et al.,* 2007; Nduka *et al.,* 2007).

#### Chemicals and reagents

Chemicals and reagents used, such as thiobarbituric acid (TBA) was procured from Sigma-Aldrich, Chemie GmbH (Steinheim, Germany), acetic-acid was from BDH Chemicals Ltd. (Poole, England), Tris-HCl buffer, sodium dodecyl sulphate, FeSO_4_, NaHCO_3_, Na_2_HPO_4_ and NaH_2_PO_4_ were of analytical grade, while the water was glass distilled.

### Lipid peroxidation assay

#### Preparation of tissue homogenates

Male rats were decapitated under mild diethyl ether anesthesia and the testis was rapidly isolated and placed on ice and weighed. This tissue was subsequently homogenized in cold saline (1/10 w/v) with about 10-up-and-down strokes at approximately 1200 rev/min in a Teflon glass homogenizer. The homogenate was centrifuged for 10 min at 3000×g to yield a pellet that was discarded, and a low-speed supernatant (SI) was kept for lipid peroxidation assay.

#### Lipid peroxidation and thiobarbituric acid reactions

The lipid peroxidation assay was carried out using the modified method of Ohkawa (Ohkawa *et al.,* 1979); a 100 µl SI fraction was mixed with a reaction mixture containing 30 µl of 0.1 M pH 7.4 Tris-HCl buffer, extract (0–100 µl). The volume was made up to 300 µl with water before incubation at 37 °C for 1h. The colour reaction was developed by adding 300 µl 8.1% SDS (sodium doudecyl sulphate) to the reaction mixture containing SI, with subsequent addition of 600 µl of acetic acid/HCl (pH 3.4) mixture and 600 µl 0.8% TBA (thiobarbituric acid). This mixture was incubated at 100 °C for 1 h. TBARS (thiobarbituric acid reactive species) produced were measured at 532 nm and the absorbance was compared with that of the standard curve using MDA (malondialdehyde).

### Data analysis

The results of the replicates were pooled and expressed as mean ± standard deviation. Student t-test, one way analysis of variance (ANOVA) and the least significance difference (LSD) were carried out. Significance was accepted at *p*<0.05 (Zar, 1984).

## Results

First, the characterization of complex mixtures of metals in municipal auto-battery leachate (MABL), Elewi Odo municipal auto-battery recycling site leachate (EOMABRL) and STREAM were investigated and the results are presented in Tables 1–3. This is to ascertain the major possible primary source of pollutants in the ambient drinking water from these batteries. The results revealed that leachate from Elewi Odo municipal auto-battery recycling site (EOMABRL) showed a high level of heavy metals such as Cu, Fe and Mn. They are higher than the permissible regulatory levels in drinking water as recommended by NAFDAC, USEPA, FEPA and WHO (Table 3). Types of battery leachate (MABL *i.e.* BL1, BL2, BL3, BL4, BL5, BL6, BL7 and BL8) showed high significant level (*p*<0.05) of heavy metals such as Cr, Pb and Cu (Tables 1 and 2). They are higher than the permissible regulatory levels in drinking water as recommended by NAFDAC, USEPA, FEPA and WHO. A similar trend was observed in water collected from the STREAM (Table 3). MABL exhibited higher levels of heavy metals than EOMABRL and the STREAM (Tables 1–3).

**Table 1:** Characterization of heavy metals in municipal auto-battery leachate (MABL) [BLI, BL2, BL3, BL4, and BL5].

	Type of battery Leachate				
Parameter	BL1	BL2	BL3	BL4	BL5
Cadmium	1.42±0.06	1.41±0.0	1.42±0.06	14.27±0.21	1.41±0.06
Cobalt	1.85±0.06	1.85±0.20	1.84±0.12	1.827±0.32	1.84±0.15
Chromium	24.10±15.59	23.47±2.50	29.53±8.51	11.30±1.35	20.43±17.62
Copper	2.78±2.41	3.28±1.88	2.44±2.63	2.44±1.33	2.18±1.06
Iron	1.77±0.40	2.32±1.01	1.76±0.36	1.83±1.73	1.73±0.15
Manganese	1.85±0.12	1.83±0.12	1.86±0.15	1.84±0.17	1.85±0.17
Molybdenum	2.17±0.45	2.01±0.20	2.03±0.15	2.07±0.92	2.00±0.20
Nickel	2.28±0.67	2.52±0.15	2.27±0.15	2.26±1.25	2.19±0.32
Lead	8.17±2.60	9.38±6.19	7.35±1.93	27.90±17.00	12.10±3.61
Selenium	2.02±0.72	2.06±0.84	2.02±0.55	2.23±3.20	2.04±0.34
Zinc	1.76±0.06	2.28±0.40	1.76±0.06	1.76±0.21	1.75±0.06

The level of heavy metals is highly significant (*p*<0.05). Values represent mean ± standard deviation, n=3. Values are measured in part per million (ppm) (Akintunde & Oboh, 2013).

**Table 2:** Characterization of heavy metals in municipal auto- battery leachate (MABL) [BL6, BL7 and BL8].

	Type of battery Leachate		
Parameter	BL6	BL7	BL8
Cadmium	1.33±1.42	1.41±0.06	1.42±0.06
Cobalt	1.85±0.42	1.86±0.21	18.67±0.06
Chromium	22.30±23.4	30.00±15.59	21.83±4.16
Copper	2.63±1.21	2.38±1.36	28.40±9.64
Iron	1.67±0.90	1.78±0.49	5.040±2.34
Manganese	1.85±0.17	1.84±0.12	1.84±0.15
Molybdenum	2.01±0.44	2.02±0.15	2.04±0.12
Nickel	2.06±2.94	2.23±0.46	2.46±0.49
Lead	10.81±8.80	5.07±0.89	7.14±2.61
Selenium	1.67±2.63	19.57±0.95	2.25±2.04
Zinc	1.80±1.39	1.90±0.15	2.92±0.52

There is a highly significant level (*p*<0.05) of heavy metals. Values represent mean± standard deviation, n=3. Values are measured in part per million (ppm) (Akintunde & Oboh, 2013).

**Table 3:** Composition of heavy metals in leachate from Elewi Odo Municipal Battery Recycling Site (EOMABRL).

	EOMABRL	STM	WHO	USEPA	FEPA	NAFDAC
**Heavy metals**	**(ppm)**	**(ppm)**	**(ppm)**	**(ppm)**	**(ppm)**	**(ppm)**
Cadmium	0.006	0.002	0.003	0.005	0.05	0.00
Cobalt	0.049	0.004	–	–	–	0.00
Chromium	0.068	0.011	0.005	0.1	0.05	0.00
Copper	0.341	0.012	–	1.0	0.3	0.00
Iron	2.66	1.076	–	0.3	0.05	0.00
Manganese	7.84	0.223	–	0.05	0.05	0.00
Nickel	0.048	0.048	–	–	–	–
Lead	0.015	1.548	0.01	0.01	0.01	0.00
Zinc	1.26	0.126	5.00	–	–	5.00

EOMABRL and STM are higher than the permissible levels of heavy metals in drinking water recommended by the World Health Organization (WHO), United States Environmental Protection Agency (USEPA), Federal Environmental Protection Agency, 1991 (FEPA), and the National Agency for Food and Drug Administration and Control (NAFDAC) (Akintunde & Oboh, 2013).

**Figure 1:**
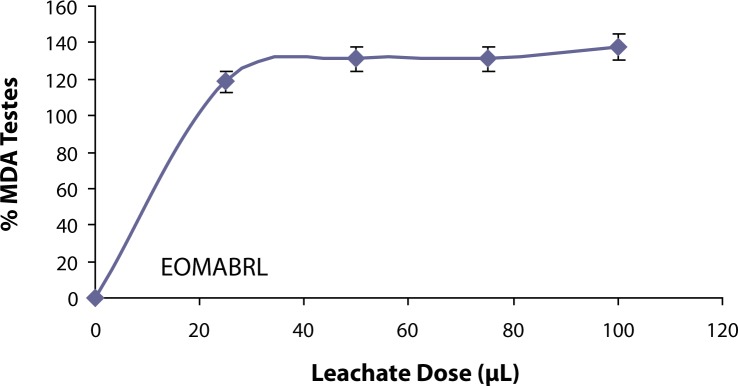
Induced lipid peroxidation in testis by leachate from Elewi Odo Municipal auto-battery recycling site (EOMABRL). TBARS (thiobarbituric acid reactive species) produced were measured at 532 nm and the absorbance was compared with that of standard using % Testis MDA (malondiadehyde). Values at all doses are significantly higher (*p*<0.05) compared with the control.

**Figure 2:**
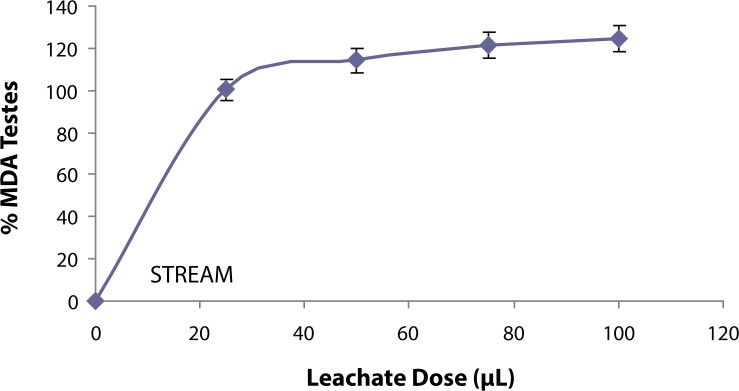
Induced lipid peroxidation in testis by STREAM water sample. TBARS (thiobarbituric acid reactive species) produced were measured at 532 nm and the absorbance was compared with that of standard using % Testis MDA (malondiadehyde). Values at all doses are significantly higher (*p*<0.05) compared with the control.

**Figure 3:**
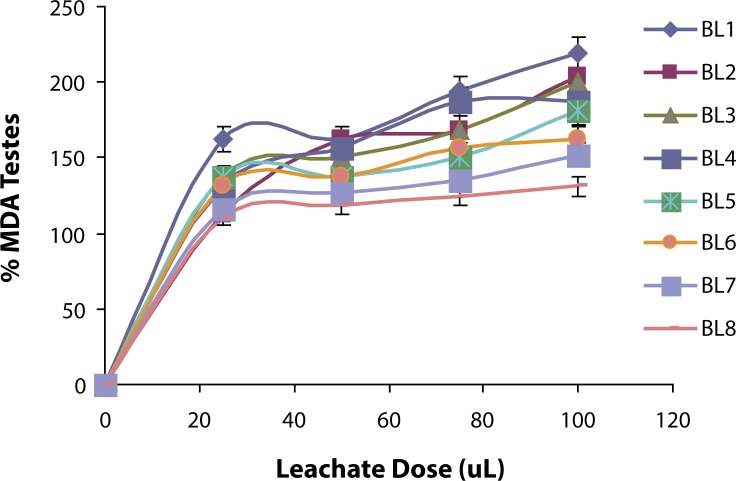
Induced lipid peroxidation in Testis by auto-battery leachate (BL1, BL2, BL3, BL4, BL5, BL6, BL7 and BL8). TBARS (thiobarbituric acid reactive species) produced were measured at 532nm and the absorbance was compared with that of standard using % Testis MDA (malondiadehyde). Values are significantly higher (*p*<0.05) compared with the control.

The ability of the leachate from Elewi Odo Municipal auto-battery recycling site (EOMABRL) on testicular lipid membrane was investigated and the *in vitro* results are presented in Figure 1. The leachate (EOMABRL) significantly (*p*<0.05) elevated testicular lipid peroxidation in a dose-dependent manner by 18.75%, 31.25%, 31.25% and 37.50% compared with the control. Similarly, the treatment of water sample from the stream close to the site (as shown in Figure 2) significantly (*p*<0.05) exacerbated the lipid products of the rat testes in a dose-dependent manner by 0.47%, 14.34%, 21.45% and 24.70% as compared with the control. Similarly, municipal auto-battery leachate (MABL) caused a significant (*p*<0.05) increase in the MDA content in a dose-dependent manner (Figure 3). MABL exhibited higher MDA products than EOMABRL and STREAM.

## Discussion

The present study suggests the induction of acidosis in mammals when leachate is ingested through any route. This is because there may be increased production of hydrogen ion by the leachates due to their inability to form bicarbonate (HCO_3_) (Seifter, 2007) and more importantly, because of the presence of acidic electrolytes in the leachates. Our observations did not support the findings of Guanagke *et al.* (2005); Farombi *et al.* (2011) who observed that leachate released from mine wastes and municipal landfill leachate were neutral. The difference may be linked to the variation of waste composition. Exposure to MABL, EOMABRL and STREAM suggests acidosis particularly during metabolic activities.

The present investigation indicated that MABL can be harmful to the testicular cell membrane on exposure and/or consumption above the bio-recommended limits. The levels of mixed-metals such as Cr, Pb and Cu were high in MABL while Cu, Fe and Mn were high in EOMABRL. Their bio-toxic effects are due to their individual, synergistic, antagonistic or collective interference with the normal testicular cell membrane. When ingested, they pass through the blood systemic circulation and are converted to their stable oxidation states (such as Pb^2+^, Cr^3+^, Cu^2+^, and Fe^3+^). These oxidized forms combine and bind with the testes bio-molecules, such as polyunsaturated fatty acids (PUFA), proteins and enzymes to form strong and stable chemical bonds. Due to their biostabilities, they become difficult to be dissociated during extraction from the body by medical detoxification therapy. This observation corroborates earlier reports where metal had been implicated as a potent inhibitor of enzymes and other macromolecules. It inhibits their functions by abstracting the hydrogen atoms from the sulphydryl groups (-SH) of cysteine and sulphur atoms of methionine (-SCH3) (Ogwuegbu *et al.,* 2003; Ogwuegbu *et al.,* 2005). Similarly, these heavy metals can replace Zn^2+^ in some dehydrogenating enzymes, *e.g.* sorbitol dehydrogenase and lactate dehydrogenase, eventually causing low motility of the sperm. Also in the process of enzyme inhibition by heavy metals, the structure of the protein molecule is mutilated to a bio-inactive form, resulting in permanent damage of the enzyme and depletion of sperm motility (Nolan, 2003; Holum, 1983; Ogwuegbu *et al.,* 2003). In addition, the level of heavy metals in MABL was higher than in EOMABRL and STREAM water. Their low levels may be attributable to the degradation and putrefaction caused by some available bacteria or fungi (acidophillus) that may be present at the site or the soil and other materials had naturally purified most of the water as it strained through the aquifer (Monroe, 2001).

Furthermore, in order to explore the possibility that the leachate and the STREAM water interfere with the structural cell membrane of the male reproductive system, the levels of malondialdehyde, *i.e.* lipid degradation products, a maker of lipid peroxidation, were evaluated in the rat testes *in vitro*. A significant dose-dependent increase was found in the levels of MDA. The higher levels of MDA in testes could be attributed to the presence of a high quantity of mixed-metals as potent activators of defective sperm-function (Pant *et al.,* 2003). They were also found to cause injury to the spermatozoa and lipid membrane (Dandekar *et al.,* 2002). Our data go along with the observation that on contact heavy metals are strongly absorbed by sperm of mammals and were reported to cause diminished sperm quality in humans and dogs (Hayes *et al.,* 1990). Spermatozoa are considered to be highly susceptible to lipid peroxidation in the presence of elevated ROS levels, due to the abundance of polyunsaturated fatty acids in their membrane (Alverez *et al.,* 1987). Increased lipid peroxidation and reduced level of antioxidant capacity of the testis in battery leachate treated rats indicated an increased free radical generation. Increased ROS formation due to lipid peroxidation and a compromised antioxidant defence system were shown to be associated with mid-piece abnormalities and decreased sperm counts (Thiele *et al.,* 1995).

Similarly, the oxidation of polyunsaturated fatty acids in biological membranes may lead to the formation and propagation of lipid radicals, uptake of oxygen, rearrangement of double bonds in unsaturated lipids, and even to destruction of membrane lipids. Many of the biochemical activities induced by these leachates can lead to the generation of products that are highly toxic to most mammalian cell types, particularly testicular cells. The present investigation confirmed that lipid peroxidation plays a significant role in the etiology of defective sperm function. Furthermore, the onset of lipid peroxidation leads to progressive accumulation of lipid hydroperoxides in the sperm plasma membrane, which decomposes to form malonaldehyde as an index of lipid peroxidative damage. It can also be predicted that loss of sperm membrane fluidity may be induced as a result of cellular injury to the spermatozoal membrane. Moreover, lipid peroxidation shown in our present data impaired the cell membrane ion-exchange, which is responsible for the normal maintenance of sperm-motility (Dandekar *et al.,* 2002, Pant *et al.,* 2003). This oxidative deterioration of polyunsaturated fatty acids results in the production of lipid radicals with ensuing formation of lipid degradation products, including malondialdehyde and other aldehydes such as alkanals, hydroxyalkenals and ketones (Selvakumar *et al.,* 2004; Schrader, 2003; Raymond *et al.,* 1998).

## Conclusion

In the present study, leachate from a municipal battery recycling site was found to be a potent source of mixed-metal exposure. These pollutants are released into the environment especially via anthropogenic sources such as industrial activities and indiscriminate dumping. They can reach underground waters, moving along water pathways and eventually they deposit in aquifers. They are equally washed away into surface waters causing subsequently water pollution. When ingested, the oxidized forms combine and bind with the testes bio-molecules, thereby leading to the accumulation of lipid hydroperoxides in the sperm plasma membrane. It decomposes to form malonaldehyde, an index of lipid peroxidative damage. In light of our results, we concluded that exposure to mixed metals from leachate obtained from the Elewi Odo municipal battery recycling site induced lipid peroxidation, impaired cell membrane, reduced sperm-membrane fluidity and caused injury to the spermatozoa in testes of male rats. Thus MABL and EOMABRL are to be considered pro-agents of testicular damage and male infertility.
